# Long-Term Outcomes Following Reconstruction of Diaphyseal Defects of the Upper and Lower Extremities Using Diaphyseal Implants: A Retrospective Study with Focus on Fixation Technique

**DOI:** 10.3390/cancers17183059

**Published:** 2025-09-19

**Authors:** Tymoteusz Budny, Anna Maria Rachbauer, Georg Gosheger, Felix Lückel, Marieke De Vaal, Sebastian Klingebiel, Jan Christoph Theil, Niklas Deventer

**Affiliations:** Department of Orthopedics and Tumororthopedics, Albert-Schweitzer-Campus 1, University Hospital Münster, 48149 Muenster, Germany; tymoteusz.budny@ukmuenster.de (T.B.); anna.rachbauer@ukmuenster.de (A.M.R.); georg.gosheger@ukmuenster.de (G.G.); felix.lueckel@ukmuenster.de (F.L.); mariekemathilda.devaal@ukmuenster.de (M.D.V.); sebastian.klingebiel@ukmuenster.de (S.K.);

**Keywords:** diaphyseal implant, biological reconstruction, bone tumor resection, sarcoma resection

## Abstract

**Background:** The reconstruction of diaphyseal bone defects following tumor resection offers various biological and endoprosthetic treatment options. The present study analyzes the impact of the fixation method (cemented; uncemented; with locking screw; without locking screw) of the diaphyseal implant on clinical outcomes. Factors such as patient age and weight as well as tumor type and location are also considered. **Methods:** This study included 39 patients who underwent intercalary endoprosthetic reconstruction of the humerus (n = 4); femur (n = 29); and tibia (n = 6) between 1998 and 2020. Prosthetic complications, fixation methods and the MSTS score for functional outcome were statistically analyzed using SPSS and R. **Results:** The event-free probability in the competing risk model was 61% (95% CI 43–74%) after one year and 11% (95% CI 3–28%) after five years. The complication rate in the patient cohort was 54%. Cementless prosthesis fixation was associated with a statistically significant better functional outcome. Additionally, higher body weight and older patient age were associated with lower MSTS scores. **Conclusions:** Patients requiring rapid remobilization or adjuvant radiation therapy may benefit more from diaphyseal implants compared to biological reconstructions. However, the complication and revision rates of diaphyseal implants are elevated. The chosen fixation method shows a statistically significant influence on functional outcome.

## Simple Summary

Reconstruction using diaphyseal implants has been insufficiently studied with regard to functional outcomes and prosthesis survival. This study included 39 patients who underwent intercalary endoprosthetic reconstruction of the humerus, femur, and tibia. It analyzes the impact of the fixation method (cemented; uncemented; with locking screw; without locking screw) of the diaphyseal implant on clinical outcomes. The event-free probability in the competing risk model was 61% (95% CI 43–74%) after one year and 11% (95% CI 3–28%) after five years. The complication rate in the patient cohort was 54%. Higher body weight and older patient age were associated with lower MSTS scores. Patients requiring rapid remobilization or adjuvant radiation therapy may benefit more from diaphyseal implants compared to biological reconstructions. However, the complication and revision rates of diaphyseal implants are elevated.

## 1. Introduction

Bone and soft tissue sarcomas as well as bony metastases of the extremities can be treated reconstructively or ablatively. Until the 1970s, amputation was the gold standard, but nowadays limb-sparing therapy is the treatment of choice. The goal of surgical therapy is complete tumor removal including the best possible oncological, functional, and cosmetic outcomes [1]. Early diagnosis and careful planning are essential for a successful procedure enabling tumor resection while preserving the joints [2]. In cases of complete resection of joint-adjacent osseous tumors, the proximal or distal joint can be removed and replaced with an endoprosthesis.

For diaphyseal defects, biological reconstruction has been used more frequently in the past. Biological reconstruction options include allograft, fibula graft (vascularized or free), combined vascularized fibula with allograft, segmental bone transport/distraction osteosynthesis, extracorporeal devitalized autograft, Masquelet technique (bone cement spacer and “induced-membrane” technique), segmental prosthesis [3]. However, these reconstruction types result in high complication rates including non-union (40%), fracture (29%), infection (14%), and generally longer periods of non-weight bearing and use of orthoses [4,5].

Therefore, diaphyseal implants represent a considerable alternative to biological reconstructions. Since purely diaphyseal implants theoretically allow joint preservation, it raises the question whether this leads to improved function and longer implant survival [6]. However, because diaphyseal malignant tumors are relatively rare and the use of mega-prosthetic diaphyseal replacements is a relatively recent approach, the available literature on clinical outcomes remains limited [7]. Over the past few years, customized 3D printing methods have been successfully used to create diaphyseal implants [8].

Due to a lack of current studies, this study investigates the functional outcome of diaphyseal implants using the MSTS score. It also examines various anchoring techniques. There is still disagreement regarding the use of bone cement and locking screws. Since aseptic prosthetic loosening is one of the most common complications, optimal fixation is crucial [9,10,11].

The study also analyzes the influence of tumor location, stem type, age, weight, and tumor size and resection length on functional outcomes, prosthetic stability and risk factors that may lead to complications. The results are compared with existing literature and evaluated against other reconstructive methods. Complication rates for femur and tibia reconstructions are compared as well as the outcome and survival of personalized (“custom-made”) versus standard (“off-the-shelf”) implants.

## 2. Materials and Methods

### 2.1. Patient Cohort

This study included 39 consecutive patients (20 female, 19 male) who were treated with diaphyseal resection due to a primary malignant bone tumor or metastasis followed by reconstruction with a diaphyseal implant at the University Hospital Muenster between 1998 and 2020 (Table 1).

The average age at the time of treatment was 55.4 years (range 10–85). Fourteen patients (35.9%) with a mean age of 48.4 years (range 21–80) were still alive at the time of data collection, while 25 patients (64.1%) had died. The average age at the time of death was 66 years (range 47–86).

The median follow-up duration for all patients was 47.6 months (range 1–145 months).

Postoperatively, 32, 25, and 12 patients survived at 1, 2, and 5 years, respectively. Among surviving patients, the mean follow-up was 70.8 months (range 17–145 months).

### 2.2. Patient Groups

Patients were assigned to different groups. In addition to the prosthesis location (femur n = 29, tibia n = 6, humerus n = 4), classification was additionally based on the anchoring mechanism (cemented vs. uncemented, screwed vs. unscrewed). A total of 64 stems were cemented, while 12 were implanted without cement. Forty stems were screwed, and 36 were not.

Twenty-eight prostheses were fully cemented. In two cases, the locking mechanism could not be determined due to lack of documentation and absence of digital X-ray images.

Furthermore, a distinction was made based on tumor type, and the cohort was subdivided according to existing internal medical conditions.

### 2.3. Methodology

**Figure 1:**
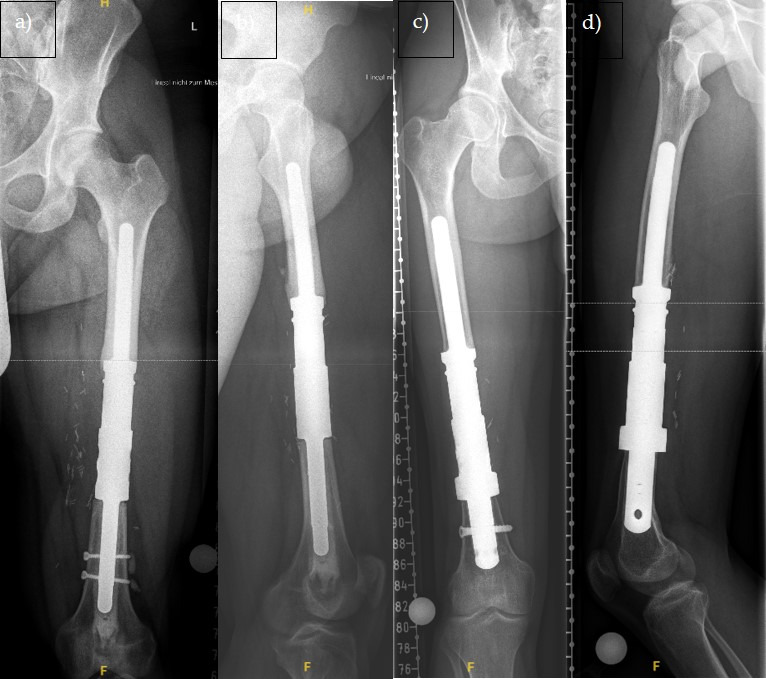
X-ray image in frontal (**a**) and lateral (**b**) view of a 34-year-old patient with a left femoral diaphysis implant (standard) and X-ray image in frontal (**c**) and lateral (**d**) view of a 26-year-old patient with a left femoral diaphysis implant with ultra short stem (custom made).

Data acquisition was conducted using keyword-based searches in the hospital information system (Orbis, Dedalus HealthCare GmbH, Bonn, Germany) and using the surgical and procedural coding system (OPS). In addition to collecting baseline data, complications and failure rates of the surgical procedures were evaluated, and radiological imaging was analyzed using the PACS viewer (Picture Archiving and Communication System; Figure 1). Prosthesis-related complications were classified according to Henderson et al. [11,12] into five types, distinguishing mechanical from non-mechanical complications.

The complications were worked up using existing medical documentation (including daily notes and surgical records) as well as patient interviews. In addition to digital data collection, patients were contacted during routine follow-up appointments at the tumor orthopedic outpatient clinic. This allowed the functional outcome to be recorded in 14 patients.

While the main endpoint of the study was prosthesis survival and complication rate depending on the anchoring mechanisms, functional outcomes were also assessed using the MSTS score (Musculoskeletal Tumor Society) [13]. Due to the retrospective character of the study, the timing of the assessment varied on a case-by-case basis during follow-up.

### 2.4. Statistical Methods

Statistical evaluations were performed using SPSS Statistics (IBM, Version 27.0.0.0), R (The R Foundation, Version 4.0.3), and Microsoft Excel (Version 16.53).

Descriptive statistics such as frequencies, means, medians, minimum, maximum, and standard deviation were calculated using SPSS.

Cross-tabulations and Pearson correlation analyses were conducted to investigate relationships such as between prosthesis location (femoral/tibial/humeral) and MSTS scores (0–30 points). Prosthesis survival required a more complex analysis due to two competing endpoints in tumor patients: prosthesis failure and death.

A “competing-risk analysis” using R was employed. In this method, patients whose follow-up was shorter than 60 months were censored.

## 3. Results

### 3.1. Surgical Indications and Comorbidities

Within the studied cohort, various underlying pathologies lead to implantation of a diaphyseal endoprosthesis. The primary surgical indication were malignant bone tumors in 24 patients and metastatic disease in 15 patients. In case of soft tissue sarcoma, tumor extension in direct contact to the bone required a diaphyseal segment resection with following reconstruction.

Patients in the primary tumor group exhibited a significantly higher five-year postoperative survival probability compared to those in the metastasis group. According to Gray’s test, the cumulative incidence of death (0 = alive, 1 = deceased) during follow-up significantly differed between the two groups (*p* < 0.05).

Table 2 and Table 3 provide detailed survival probabilities at 1, 2, and 5 years, stratified by individual diagnoses.

Additional influencing factors on overall patient survival included a variety of pre-existing and comorbid medical conditions. The most common comorbidities were internal medical diseases such as arterial hypertension and diabetes mellitus.

### 3.2. Event-Free Probability

This study defined two competing endpoints for the observation period: prosthesis failure—either due to revision surgery or prosthesis explantation—and death prior to prosthesis failure. Event-free probability was calculated as the time interval during which neither event occurred.

Despite high complication rates following implantation, prosthesis survival was frequently limited. Contributing factors included aseptic and septic loosening as well as periprosthetic fractures [14].

The Event-free probability in the competing risk model, considering “prosthesis failure” and “death” as competing events, was:

61% (95% CI 43–74.0%) at 12 months,
26% (95% CI 13–41%) at 24 months,
11% (95% CI 3–28%) at 60 months.

In 18 of 39 cases (46%), prosthesis explantation was necessary. The median time to explantation was 12 months postoperatively (interquartile range: 5–18 months). In five patients (13%), diaphyseal prosthesis implantation was ultimately followed by limb exarticulation or amputation.

### 3.3. Implant Complications: Types and Timing

Prosthesis-related complications were classified into five categories per Henderson et al. [12]. Table 4 shows the types, frequencies, anatomical locations, and average time points of complication onset.

Six patients experienced a second complication following the initial revision. Four were cases of recurrent aseptic loosening (Type 2), and two involved local tumor recurrence (Type 5). In one instance of recurrent Type 2 failure, an additional Type 3 complication—a periprosthetic fracture—occurred.

Aseptic loosening had an average onset of approximately 10 months postoperatively. The anatomical distribution included five femoral, two tibial, and one humeral implant (Table 5).

Of the four prosthetic infections (Type 4 complications), two affected femoral and two tibial prostheses. No consistent microbial pattern emerged. In one case, Streptococcus intermedius infection necessitated high-leg exarticulation. Table 6 shows prosthesis failure depending on the underlying diagnosis.

### 3.4. Risk Factors for Implant Complications

**Figure 2:**
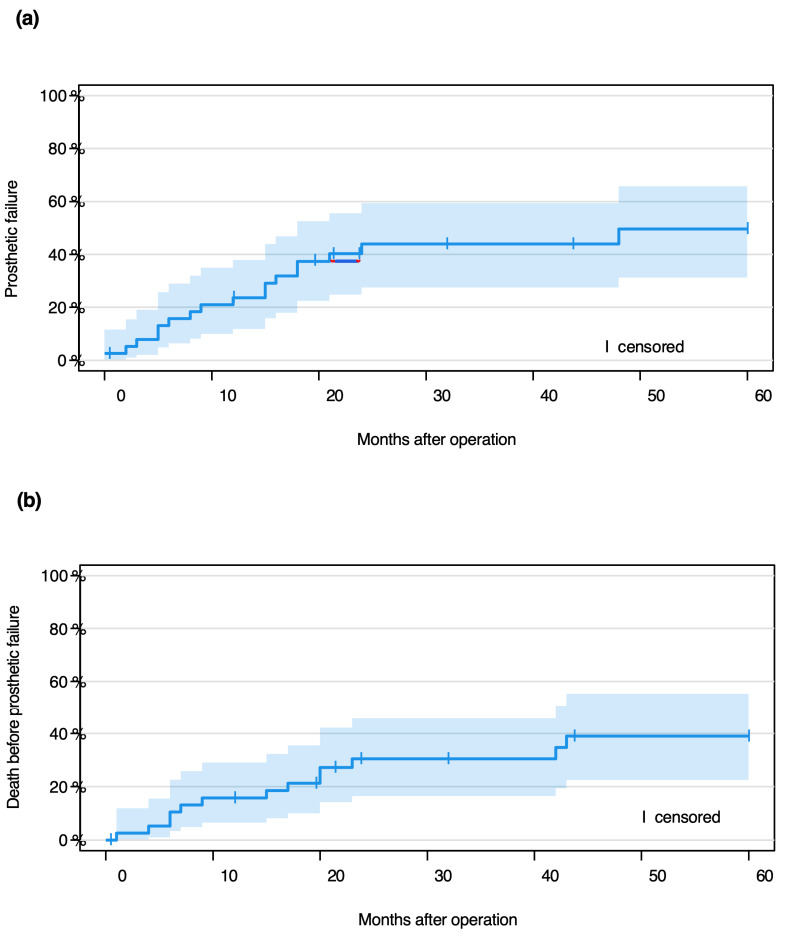

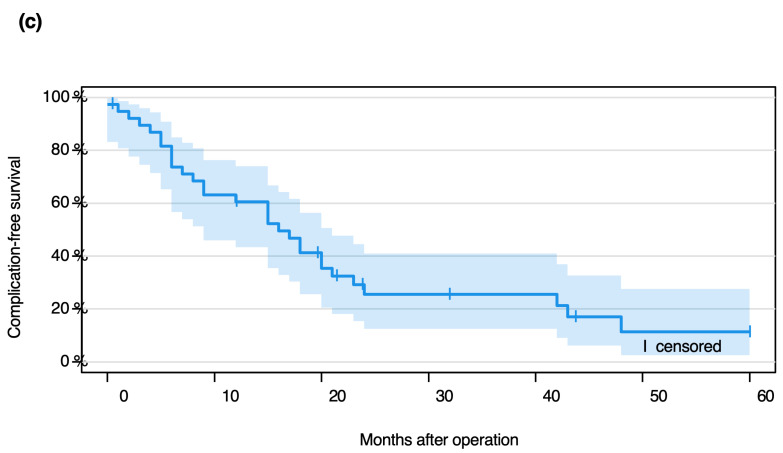
Cumulative incidence function with log-log transformed pointwise confidence intervals: probabilities of prosthetic failure, death before prosthetic failure and complication-free survival. Patients whose follow-up was shorter than 60 months were censored. (**a**) probability of prosthetic failure; (**b**) probability of death before prosthetic failure; (**c**) probability of complication-free survival.

Fine Gray analysis (Figure 2) was used to determine event-free probabilities over one and five years, including both point-wise and log-log transformed confidence intervals. Although certain trends were observed in sub-hazard ratios, no statistically significant risk factors for revision-free survival could be identified. Radiotherapy in particular showed no significant effect on revision free survival. Missing data for variables such as BMI, prior radiation, and chemotherapy limited interpretability in some subgroups (Table 7).

Competing risk analysis showed that neither weight (kg) nor age (years) significantly affected the risk of prosthesis failure. However, increased age was significantly associated with higher risk of death (*p* < 0.01).

A statistically significant association was found between gender and prosthesis failure within the first postoperative year (Fisher’s Exact Test, *p* < 0.05), with an odds ratio of 6.9 (95% CI 1.1–42.8), indicating a 6.86-fold increased risk in male patients. This association was not statistically significant at the two- or five-year mark. Gender also significantly influenced prosthesis survival time (*p* < 0.05). A Cohen’s d of −0.6 indicated a medium effect size, with female patients demonstrating longer prosthesis survival. Although women showed a higher probability of Event-free probability up to five years, the difference was not statistically significant (*p* = 0.11). Men had a higher average BMI and body weight at surgery: Mean total weight: 80 kg (SD = 23.6), females: 72 kg (SD = 24.0), males: 90 kg (SD = 19.7).

No significant correlation was observed between BMI and survival at 1, 2, or 5 years. Likewise, a higher BMI (≥25) was not significantly associated with mortality during the study (Fisher’s Exact Test, *p* = 0.14). Odds ratio = 4.000 (95% CI: 0.9–18.2). BMI also had no significant effect on revision-free survival.

The primary focus of this study was on the influence of fixation technique on functional outcome and implant survival. Among 78 stems:

• 35 were cemented and screwed;
• 29 were cemented without screws;
• 5 were screwed but not cemented;
• 7 were neither cemented nor screwed;
• 1 case lacked fixation documentation.

Of 39 prostheses, eight failed due to aseptic loosening (20.5%). Loosening affected:

• 7 of 64 cemented stems (11%);
• 1 of 12 uncemented stems (8%);
• 3 of 40 screwed stems (8%);
• 5 of 36 non-screwed stems (14%).

Loosening occurred equally in proximal and distal stems (4 each; Table 7). No statistically significant correlation was found between fixation type and loosening (Fisher’s Exact Test), although prostheses with cemented, non-screwed fixation showed a higher (but not significant) tendency toward loosening (odds ratio: 3.1, 95% CI: 0.7–13.9).

In total, 21 of 39 prostheses (54%) were affected by Henderson-classified complications. The sample size limited further subgroup analyses.

Among the 78 stems:

• 14 (18%) were custom-made;
• 29 (37%) were off-the-shelf.

A significant association was found between stem type and aseptic loosening (*p* < 0.05), with an odds ratio of 5.4 (95% CI: 1.0–28.9), indicating a higher risk with custom-made stems. Custom stems predominantly had a six-sided solid metal design (13 of 14); one used a “hollow cage” design, which remained complication-free.

The average resection length was 15.4 cm (SD = 4.5), varying by location:

• Femur: 16.4 cm (SD = 4.6);
• Tibia: 14.8 cm (SD = 3.1);
• Humerus: 8.6 cm (SD = 1.0).

### 3.5. Functional Outcome

The MSTS score was recorded in 25 patients. Across all fixation types, the median MSTS score was 15.0 (IQR: 9.5–25.5).

• Cemented: median 14.5 (IQR: 8.0–25.0);
• Uncemented: median 27.0 (IQR: 17.0–29.0);
• Screwed: median 15.5 (IQR: 10.3–25.3);
• Non-screwed: median 14.5 (IQR: 10.3–25.3).

Cemented prostheses had significantly lower MSTS scores (*p* < 0.05, r = −0.39). Screwing status showed no statistically significant difference.

By location:

• Femoral: median 15.5 (IQR: 8.8–25.8);
• Tibial: median 17.5 (IQR: 9.0–25.3);
• Humeral: 14.0.

A significant moderate negative correlation was found between body weight and MSTS score (Pearson r = −0.4, *p* < 0.05, CI: −0.7 to −0.03).

Age also negatively correlated with MSTS outcome (Pearson r = −0.6, *p* < 0.001, CI: −0.817 to −0.303). Patients with metastases were significantly older than those with primary tumors (Cohen’s d = −1.3, *p* < 0.05).

Gender did not significantly influence MSTS scores.

Patients with primary tumors had significantly higher MSTS scores than those with metastases (*p* < 0.05, r = 0.4).

• Primary tumor: median 20.5 (IQR: 12.5–26.8);
• Metastasis: median 8.0 (IQR: 6.5–20.5).

Internal medical comorbidities were associated with significantly lower MSTS scores (*p* < 0.05, r = 0.47).

• Without comorbidities: median 25.0 (IQR: 16.0–28.0);
• With comorbidities: median 13.0 (IQR: 8.0–16.5).

Orthopedic, psychological, or neurological comorbidities did not significantly affect functional outcomes.

Radiation, neoadjuvant chemotherapy, and adjuvant therapy showed no significant impact on MSTS scores.

## 4. Discussion

### 4.1. Diaphyseal Implants Have Higher Complication Rates Than Biological Reconstructions

Multiple surgical techniques are used for diaphyseal reconstruction post-tumor resection, with no consensus on the ideal method [14,15,16,17]. Lun et al. [17] compared segmental allografts (n = 18) to intercalary prostheses (n = 16) and found significantly lower complication rates in the prosthesis group (19% vs. 67%) along with faster mobilization.

Biological methods like vascularized fibula with allografts offer good integration especially in younger patients, whereas isolated allografts have poor healing due to lack of vascularization [18]. Errani et al. reported a 30% complication rate in combined reconstructions, which is better than isolated allografts [16].

In the literature, complication rates for intercalary prostheses are reported with a range from 14% to 50% [9,15]. Streitbürger et al. described 21 complications in 28 prostheses, representing an even higher rate [14]. Low complication rates were primarily reported by Huang et al. [9]; however, it is important to emphasize that their study involved a small patient group of only 16 patients, of whom 14 had already died within two years. Benevenia et al. reported on a larger cohort of 41 patients [15]. In both studies, complications appear to have occurred relatively early after surgical treatment, as the median follow-up was only 19 and 11 months, respectively. In comparison, the present study shows a complication rate of 54% observed in 39 patients with a median follow-up of 47.6 months.

Overall, the differences between the individual study groups are likely attributable to the varying patient numbers and the differences in follow-up duration. Though prostheses enable quicker recovery, Yao et al. reported good long-term functional outcomes with intercalary allografts (MSTS 26.2) [19].

Biological methods require long unloading periods and provide limited primary stability [17,20]. Nevertheless, biological reconstructions continue to play an important role in the treatment of bone defects. Diaphyseal prostheses are preferred for patients with limited life expectancy or chemotherapy needs due to faster rehabilitation [6, 7].

Although diaphyseal implants are associated with faster recovery, there is no conclusive evidence that they result in higher complication rates than biological options [14,15,16,17,18].

### 4.2. Cement and Screw Fixation Reduces Aseptic Loosening Risk

Diaphyseal implants allow for cemented or uncemented fixation with optional screw support. Factors like age, bone quality, smoking, and comorbidities influence the choice of procedure. Cementation is advised for compromised bone [15], while younger patients often receive uncemented stems [18].

In our study, cemented stems with screws had a 6% loosening rate, compared to 20% in uncemented stems with screws. However, the uncemented group was small. Benevenia et al. [15] found better MSTS scores (*p* < 0.01) and fewer complications with cemented stems (21%) than uncemented (33%, *p* = 0.390). However, it should be mentioned that the follow-up periods in the study of Benevenia et al. and the present study differed significantly (11 vs. 47.6 months). This may affect the rate of aseptic loosening significantly. Contradictory, our uncemented group had better function, likely due to younger age and less adjuvant radiation therapy.

Additionally, antibiotic-loaded cement also helps to prevent infection and recurrence [21]. Though not statistically significant, cemented stems with screws failed less often (5.7%) than cemented stems without screws (17.2%).

### 4.3. Prosthesis Failure Is More Likely After Primary Tumor Resection, but Not Significantly

The goal of reconstruction is long-term, complication-free function. In our cohort, implant failure rates were: 1-year: 25.0%, 2-year: 52.0%, 5-year: 75.0%.

Survival varies by malignancy. Patients with secondary tumors had significantly higher mortality (Gray’s Test, *p* < 0.05). Survival in primary tumor patients was higher: 1Y: 87.0%, 2Y: 73.9%, 5Y: 47.1% vs. metastasis patients: 1Y: 73.3%, 2Y: 53.3%, 5Y: 28.6%. These findings align with Pu et al. [22] and Hanna et al. [10].

Patients with soft tissue sarcomas who underwent diaphyseal resection demonstrated higher rates of postoperative prosthetic failure. This may be attributed to inadequate soft tissue coverage of the implant and the high incidence of postoperative radiotherapy (73%).

Despite longer survival in primary tumor patients, prosthetic failure rates at 1, 2, and 5 years showed no significant difference between groups (Fisher Exact Test). One-year revision-free survival was slightly better in primary tumors (65% vs. 53%), but this narrowed by year five (12% vs. 9%).

Thus, tumor type does not significantly affect prosthesis survival, supporting use in both groups.

### 4.4. Internal Comorbidities Affect Function More than Survival

Patients with internal diseases had a higher mortality rate (73% vs. 53%), but this was not statistically significant (*p* = 0.31). However, internal diseases significantly worsened functional outcomes (MSTS: 13 vs. 25.), likely due to impaired rehabilitation.

Comorbidities can also impact cancer progression. Stelzl et al. linked diabetes to worse soft tissue sarcoma survival (*p* < 0.05) [23], possibly due to insulin/IGF-1 effects. Qu et al. identified low HDL-C as a negative prognostic marker [24].

While internal diseases may shorten survival and reduce complication risk due to limited prosthesis lifespan, they also hinder functional outcomes—making faster-recovery implants preferable.

### 4.5. Overweight Patients Have Worse Function but No Increased Failure Risk

The average BMI in our adult cohort was 28.1, classifying most as overweight [25]. Excess weight increases mechanical load on implants, especially in lower limbs, and may raise the risk of loosening [25,26]. Yet, some long-term studies found no correlation between BMI and prosthetic failure [12,27].

Functionally, we observed a significant inverse relationship between body weight and MSTS score (r = −0.42, *p* < 0.05; 95% CI: −0.70 to −0.03), consistent with previous findings [28]. However, no significant link was found between BMI and prosthesis survival.

Obesity-associated conditions like depression and fatigue may also impair function [29], but BMI alone shouldn’t contraindicate diaphyseal implants.

### 4.6. Functional Outcomes in Diaphyseal Implants

We recorded MSTS scores in 25 patients only: 20 femoral (median: 15.5), 4 tibial (17.5), and 1 humeral (14). Limited humeral data restricts conclusions. However, the poorer functional outcomes observed in patients with secondary cancer may be attributed to the gradual deterioration of their general health status. Moreover, long-term survival in this group is significantly reduced, which may prevent the occurrence or detection of late-onset complications. External studies show promising results. Pu et al. reported an MSTS of 28.6 in humeral implants [22], and Zhao et al. found 27.2 (primary) and 26.1 (metastases) over 45 months [30].

Benevenia et al. reported average MSTS scores of 24.9 (humerus), 22.5 (femur), and 23.1 (tibia) [16], with no complications in humeral or tibial implants. Conversely, Mahdal et al. reported a 44% complication rate in humeral implants [31].

Huang et al. reported an MSTS of 25.4 in femoral implants [11]. Despite limited humeral data in our study, the literature supports their functional viability

### 4.7. Tibial Implants Have Higher Failure Rates than Femoral Ones

Tibial implants had a median MSTS of 17.5 but high complication rates. Of six patients, 33.3% had infections or loosening, and 83.3% experienced prosthesis failure—compared to 37.9% in femoral implants.

Poor soft tissue coverage in the tibia, often addressed via gastrocnemius flaps, may increase infection risk [31,32,33,34]. Given this, primary amputation or biological reconstruction (e.g., vascularized tibia, distraction osteogenesis) may be considered [35,36,37,38,39,40].

Stem modifications like “ultra-short stems” could reduce loosening risk [41], though more robust data is needed.

### 4.8. Custom-Made Stems Are Linked to Worse Outcomes

In this study, 14 (17.9%) custom-made stems and 29 (37.2%) off-the-shelf stems were analyzed. Statistical analysis revealed that 71.4% of prostheses with a custom-made stem experienced prosthetic failure, compared to 46.8% of those without a custom-made stem. Fisher’s exact test demonstrated a statistically significant association between the implantation of a custom-made stem and the event of aseptic loosening. The odds ratio of 5.4 (95% CI: lower 1.04; upper 28.9) suggests that the risk of aseptic loosening is increased by a factor of 5.4 in custom-made stems compared to standard stems. However, it should be noted that the wide confidence interval limits the interpretability of the odds ratio.

Thirteen of the custom-made stems were so-called hexagonal solid metal stems, while one was a “hollow cage stem” consisting of a lattice structure. Complications occurred exclusively in the hexagonal solid metal stems. It is important to consider the uneven distribution between hollow cage and solid metal stems in this context [15,41].

Anchorage under 5 cm compromises stability [42]. While resection length often predicts failure, we found no such link. “Ultra-short stem” systems introduced in 2017 for short anchorage tibial reconstructions showed good results [41].

3D-printed implants also show promising results: Zhao et al. reported early integration and improved function in tibial reconstructions [43], and Shao et al. demonstrated success in femoral applications [8]. Bischel et al. found conical stems suitable for large defects and hexagonal stems better for distal areas [44]. Nevertheless, studies should consider not only the length of the prosthetic stem but also, in particular, the impact of its diameter

### 4.9. Study Limitations

This study spans patients from 1998 onward to include long-term outcomes. But prosthesis designs, surgical techniques and adjuvant chemotherapy/radiation protocols have evolved substantially from 1998 to 2020. Therefore, the comparability of cases between 1998 and 2020 is limited. Additionally, the use of custom versus off-the-shelf implants followed clinical judgment, which may confound outcome comparison. The rarity of diaphyseal tumors limited cohort size and statistical power, especially for humeral and tibial cases. Anchorage techniques were unevenly distributed across anatomical sites.

Additionally, the fatal nature of underlying malignancies led to patient loss during follow-up. Mortality-related cohort reduction must be considered when interpreting prosthesis survival rates at 1, 2, and 5 years.

MSTS scores were assessed retrospectively postoperatively as part of the study. In total, follow-up contact could be established in 25 cases only, as some patients had passed away. The timing of this assessment varied on a case-by-case basis.

## 5. Conclusions

Diaphyseal implants can, in cases without complications, allow for preservation of the affected limb and a rapid return to everyday life. However, this study cohort showed that complication and revision rates are elevated with intercalary prostheses. The following findings can be derived from this study on the long-term outcomes of diaphyseal implants:

The probability of event-free probability after five years is low at 11.4% (95% CI 2.5–27.5%).

- The fixation method significantly influences the outcome of the implants.
- The risk of aseptic loosening tends to be lower for cemented and screwin stems than for cemented unscrewed stems. However, this result is not statistically significant.
- Risk factors that can negatively influence the functional outcome in a statistically significant manner include increased body weight, advanced patient age, and preexisting internal medical conditions.

Therefore, the use of diaphyseal implants should be carefully considered on a case-by-case basis, taking into account the risk factors mentioned above and the experience of the surgeons. To answer the question of the ideal fixation technique and to build a larger database on prosthesis survival times, functional outcomes, and best surgical techniques, further medium- to long-term studies on diaphyseal implants are needed. So far, cemented stems, that are locked with additional screws, seem to be the best option.

## Figures and Tables

**Table 1:** Overview of the patient cohort.

No	Age	Diagnosis	Localiza tion	Bone/Soft Tissue	Seize	Enneking Classification	Prior Operation	Chemo	Response	Radiation	FU
1	26	Extraskelettal	Femur	ST	5–10	IIa	-	Y	2	N	NED 42
		OS									
2	63	OS	Femur	Bone	>10	IIb	-	Y	2	N	DOUC
											76
3	16	EWS	Femur	Bone	5–10	IIa	-	Y	1	N	NED 54
4	32	Extraskelettal EWS	Femur	ST	5–10	na	-	Y	1	N	NED 24
5	35	Soft Tissue Sarcoma	Femur	ST	5–10	IIb	-	Y	Na	N	NED 76
6	59	Soft Tissue Sarcoma	Femur	ST	5–10	IIb	-	Y	Na	Y	AWD 32
7	59	Soft Tissue Sarcoma	Femur	ST	5–10	IIa	-	Y	Na	Y	AWD 91
8	80	Soft Tissue Sarcoma	Femur	ST	5–10	IIb	-	N	-	Y	DOD 4
9	55	MFH	Femur	ST	>10	IIa	Intralesional. Resection	N	-	N	DOD 17
10	48	Soft Tissue Sarcoma	Femur	ST	5–10	IIa	Intralesional Resection	N	-	Y	DOD 10
11	59	Soft Tissue Sarcoma	Femur	ST	5–10	IIb	Intralesional Resection	Y	Na	Y	DOUC 6
12	53	Soft Tissue Sarcoma	Femur	ST	>10	IIa	Marginal Resection	N	-	Y	NED 97
13	41	Soft Tissue Sarcoma	Femur	ST	>10	IIa	Intralesional Resection	Y	Na	Y	AWD 104
14	52	Soft Tissue Sarcoma	Femur	ST	>10	IIb	-	Y	Na	Y	DOD 20
15	54	Soft Tissue Sarcoma	Femur	ST	>10	IIa	-	Y	Na	Y	DOUC 42
16	69	Renal-CA	Femur	Bone	5–10	Met	-	N	-	N	AWD 28
		Metastasis									
17	62	Renal-CA	Femur	Bone	<5	Met	Intramed	N	-	Y	DOD 80
		Metastasis					Nailing				
18	62	Renal-CA	Femur	Bone	5–10	Met	Intramed	N	-	N	DOD 9
		Metastasis					Nailing				
19	58	Renal-CA Metastasis	Femur	Bone	5–10	Met	Plate-Osteosynthesis (ORIF)	N	-	N	DOD 43
20	60	Renal-CA	Femur	Bone	<5	Met	-	N	-	Y	DOD 98
		Metastasis									
21	79	Renal-CA	Femur	Bone	<5	Met	-	N	-	N	DOD 1
		Metastasis									
22	62	CS	Femur	Bone	Na	IIa	Intralesio-	Y	Na	N	DOD 23
							nal Cur-				
							retage				
23	72	NSCLC Meta-	Femur	Bone	<5	Met	-	N	-	Y	DOD 6
		stasis									
24	85	Breast-Ca	Femur	Bone	<5	Met	-	Na	-	Y	DOD 7
		Metastasis									
25	82	Breast-Ca	Femur	Bone	5–10	Met	-	N	-	Y	DOD 49
		Metastasis									
26	17	OS	Femur	Bone	>10	IIb	-	Y	2	N	NED 44
27	10	EWS	Femur	Bone	5–10	IIb	-	Y	2	N	AWD
											144
28	60	Soft Tissue Sarcoma	Femur	ST	>10	IIa	-	N	-	Y	DOD 48
29	69	Renal-CA	Femur	Bone	5–10	Met	Intramed	Y	Na	Y	DOD 17
		Metastasis					Nailing				
30	54	Soft Tissue Sarcoma	Tibia	Bone	<5	IIa	-	N	-	N	AWD 6
31	44	NOS	Tibia	Bone	5–10	IIb	-	Y	2	N	DOD 32
32	47	NOS	Tibia	Bone	>10	IIb	-	Y	Na	N	DOD
											102
33	59	Renal-CA	Tibia	Bone	>10	Met	-	Y	Na	Y	DOD 73
		Metastasis									
34	44	Adamantinoma	Tibia	Bone	5–10	IIb	-	N	-	N	AWD
											114
35	60	Colon-CA Metastasis	Tibia	Bone	5	Met	-	Na	-	Y	DOD 54
36	76	Soft Tissue Sarcoma	Humerus	ST	5–10	IIb	-	Y	Na	Y	NED 50
37	68	Renal-CA	Humerus	Bone	Na	Met	-	Na	-	Na	DOD 81
		Metastasis									
38	64	NSCLC Meta-	Humerus	Bone	<5	Met	-	N	-	N	DOD 20
		stasis									
39	66	Breast-Ca	Humerus	Bone	5–10	Met	-	N	-	Y	DOD 15
		Metastasis									

No: Number, Age on the day of surgery (years), MFH: Malignant fibrous, ST: Soft Tissue, Seize in cm, Na: Not available, Met: Metastasized, IIA: Intracompartimental tumor extension, IIB: Extracompartimental extension, Y: yes, N: no, Response: Response to chemotherapy according to Salzer-Kuntschik, FU: Follow-up in months, DOD: death of disease, DOUC: death of unknown cause, NED: no evidence of disease, AWD: alive with disease.

**Table 2:** Cumulative 1-, 2-, and 5-year patient survival in relation to diagnosis: primary tumor or metastasis.

	1-Year Survival	2-Year Survival	5-Year Survival
Overall	82%	66%	39%
Primary tumor	87%	74%	47%
metastasis	73%	53%	29%

**Table 3:** Cumulative 1-, 2-, and 5-year survival in relation to diagnosis.

Diagnosis	Number of Patients	1-Year Survival	2-Year Survival	5-Year Survival
Osteosarcoma	3	100%	100%	100%
Ewing sarcoma	3	100%	100%	100%
Soft tissue sarcoma	16	80%	67%	38%
Other primary tumor	2	100%	50%	50%
Renal cancer metastasis	9	77.8%	66.7%	50%
Pulmonary cancer metastasis	2	50%	0%	0%
Breast cancer metastasis	3	67%	33%	0%
Colrectal cancer metastasis Metastase	1	100%	100%	0%

**Table 4:** Type of complication according to Henderson et al. [12] and timing of occurrence, categorized by prosthesis location.

Complication According to Henderson	Total Number of Cases (n = 21)		Femoral (n = 13)	Tibial (n = 5)	Humeral (n = 3)	Time in Months After Implantation Mean (SD)
**Type 1**	1	5%	0	1	0	3 (-)
**Type 2**	8	38%	5	2	1	10 (6)
**Type 3**	2	10%	2	0	0	17 (2)
**Type 4**	4	19%	2	2	0	8 (11)
**Type 5**	4	19%	3	0	1	22 (18)
**unclear**	2	10%	1	0	1	6 (9)

**Table 5:** Number of prostheses by location and percentage of aseptic loosening and total prosthesis explantations.

Localization	Number of Protheses	Aseptic Loosening		Total Prosthesis Explantation	
Femoral	29	5	17%	11	38%
Tibial	6	2	33%	5	83%
Humeral	4	1	25%	2	50%

**Table 6:** Prosthesis failure during lifetime depending on diagnosis.

Diagnosis	Number of Prostheses (n = 39)	Prosthesis Failure During Lifetime	Prosthesis Failure During Lifetime in %
Osteosarcoma	3	1	33%
Ewing-Sarcoma	3	2	67%
Soft Tissue Sarcoma	16	9	56%
Renal Cell Carcinoma Metastasis	9	3	33%
Other Primary Tumor	2	1	50%
Lung Cancer Metastasis	2	0	0.0%
Breast Cancer Metastasis	3	1	33%
Colon Cancer Metastasis	1	1	100%

**Table 7:** Relative Loosening Rate of Individual Shafts Depending on the Fixation Mechanism.

Fixation Mechanism	Number of Stems	Number of Cases with Aseptic Loosening	Loosening Rate
cemented+ screwed	35	2	6%
cemented+ unscrewed	29	5	17%
uncemented + screwed	5	1	20%
uncemented + unscrewed	7	0	0%
No information	2	0	0%

## Data Availability

The datasets used and/or analyzed during the current study are available from the corresponding author on reasonable request.
